# Public Support for Conserving Bird Species Runs Counter to Climate Change Impacts on Their Distributions

**DOI:** 10.1371/journal.pone.0101281

**Published:** 2014-07-01

**Authors:** Thomas Hedemark Lundhede, Jette Bredahl Jacobsen, Nick Hanley, Jon Fjeldså, Carsten Rahbek, Niels Strange, Bo Jellesmark Thorsen

**Affiliations:** 1 Department of Food and Resource Economics and Centre for Macroecology, Evolution and Climate, University of Copenhagen, Copenhagen, Denmark; 2 Department of Geography and Sustainable Development, University of St. Andrews, St. Andrews, Scotland, United Kingdom; 3 Natural History Museum of Denmark and Centre for Macroecology, Evolution and Climate, University of Copenhagen, Copenhagen, Denmark; Universidad Andres Bello, Chile

## Abstract

There is increasing evidence that global climate change will alter the spatiotemporal occurrences and abundances of many species at continental scales. This will have implications for efficient conservation of biodiversity. We investigate if the general public in Denmark are willing to pay for the preservation of birds potentially *immigrating* and establishing breeding populations due to climate change to the same extent that they are for native species populations currently breeding in Denmark, but potentially *emigrating* due to climate change. We find that Danish citizens are willing to pay much more for the conservation of birds currently native to Denmark, than for bird species moving into the country – even when they are informed about the potential range shifts associated with climate change. The only exception is when immigrating species populations are under pressure at European level. Furthermore, people believing climate change to be man-made and people more knowledgeable about birds tended to have higher WTP for conservation of native species, relative to other people, whereas their preferences for conserving immigrant species generally resembled those of other people. Conservation investments rely heavily on public funding and hence on public support. Our results suggest that cross-country coordination of conservation efforts under climate change will be challenging in terms of achieving an appropriate balance between cost-effectiveness in adaptation and the concerns of a general public who seem mostly worried about protecting currently-native species.

## Introduction

The accumulating evidence of ongoing climate change has spurred increased concern about the future distribution of species. Research shows that climate change may significantly change the geographical distribution of species and life zones [1]–[7]. While the methodological basis for reliable predictions is still under development [8], there is considerable evidence that many species groups have adjusted their distributions and phenologies in response to climate change [9], [10]. Although some changes may not threaten the survival of a species, there is a growing concern that some may be pushed out of the geographical area where suitable habitats exist [4], [8]. For such species, the potential for geographical range shifts have already been hampered by the loss of natural habitats [3], [4]. Thus, conservation management is under pressure to come up with cost-efficient strategies for handling this additional complexity as re-iterated at the Nagoya convention [11]. Successful conservation programmes, however, do not only rely on their cost-effectiveness but also on popular support for public spending [12]. Hence, investigating the willingness of citizens to support national, as well as international, conservation programmes in the context of climate-induced shifts in species ranges is an important matter.

**Figure 1:**
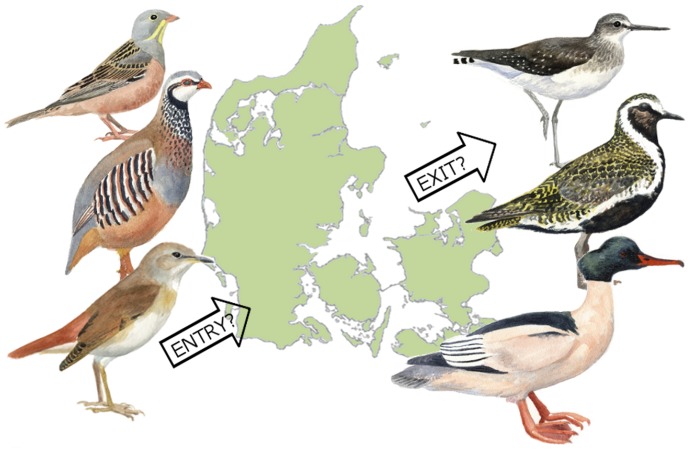
The policy challenge. The figure illustrates the overall challenge for conservation management, using the example of Denmark: Species currently breeding in an area may emigrate in the future as climate change alters habitats, whereas others may immigrate and settle for the same reason – provided suitable habitat is available.

Biodiversity conservation results in various economic benefits, including significant non-use (existence) values [13]. Most valuation studies of biodiversity apply stated preference methods, as these are able to include non-use values [14]. Such studies have documented the willingness of people to pay for the preservation of biodiversity, particularly for endangered species [15]–[17]. This study applies a category of stated preference methods known as choice modelling, which is based on the Random Utility Model [18]. Using realistic scenarios of global climate change impacts on birds and their conservation status as seen in a Europe-wide context [19] for constructing the choice exercise, we investigate whether the general public in Denmark is equally willing to pay for the conservation of climate-induced immigrating breeding birds as they are for native species currently breeding in their country, but likely to emigrate due to climate change. The concept of this policy challenge facing conservation management is illustrated in Fig. 1.

## Materials and Methods

### The case and its presentation to respondents

The future population levels of both currently-native and potentially-immigrating bird species will depend on policies implemented to protect existing habitats, to ensure development of new suitable habitats or policies that in other ways will mitigate effects of climate change on native species, and/or support the immigration and establishment of new species in Denmark. It is this choice among such alternatives that we ask respondents to indicate their preferences over.

As a team of economists and biologists we together selected at set of relevant bird species predicted to experience range shifts due to climate change and grouped them according to the nature of these changes and their current conservation status. The groups of bird species (which are the attributes of the choice alternatives) varied in several ways: *i*) whether they were native to Denmark or potentially could immigrate to Denmark due to climate change; *ii*) in their current and predicted future conservation status in Denmark; *iii*) and their current and predicted future conservation status in Europe. From early pilot studies we learned that only the very common or charismatic species were generally recognised by the public and as far as possible birds of equal charisma and level of recognisability were chosen to represent the groups of native and immigrating species. Colour drawings were specially developed for the purpose in a manner which harmonised pictorial impressions as far as was deemed reasonable.

The questionnaire was then tested thoroughly by means of individual interviews and focus group meetings involving a selection of different people from the Danish population. Before the final implementation we launched a pilot data collection. These measures enhanced the design quality and confirmed that the choice problem as presented was easily understood by laypeople.

**Figure 2:**
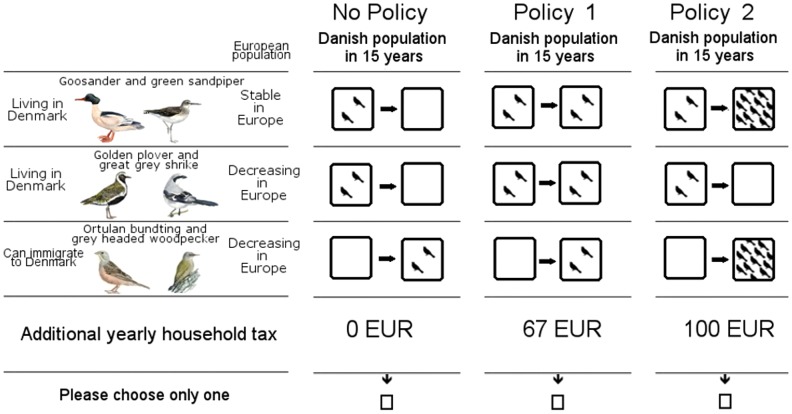
Estimating preferences for distribution changes. Here we show one example of how the decision situation was presented to respondents as a choice between policies, each respondent answering several such choice sets. The pictograms illustrating bird groups (not living in Denmark or extirpated,scarce or abundant) were explained to respondents before completing the choice tasks.

In the survey respondents were carefully informed about the predicted environmental changes for bird populations and options to remedy such effects, before being faced with six choice sets, where alternative policies would result in different bird population levels in the future (15 years ahead) for both native and immigrating species. Figure 2 shows an example of one of the choice sets presented to respondents.

There are many potential confounding concerns at play in a scenario like the present, and we carefully handled these in the questionnaire. For example, we alerted respondents to the fact that immigrating species were not to be confused with invasive species. Respondents' knowledge about birds could also play a role in their choices, and therefore we included a small quiz on bird recognition in order to control for such knowledge effects. This involved presenting them with a drawing of a bird and a limited set of alternative names to choose from. Furthermore, we asked a range of follow-up questions on their beliefs and viewpoints on climate change causes, policies and impacts.

### Ethics Statement

The respondent data were collected through the internet. Respondents were drawn from an internet panel consisting of a representative and voluntary sample of the Danish population. The panel is operated by the research institute Analyse Danmark, who abide to the ICC/ESOMAR International Code on Market and Social Research [20] that ensures an ethical research practice. For this specific survey, respondents were introduced to the topic of the survey on the first page and could withdraw if they did not want to answer, thus consent is assured in addition to that given to Analyse Danmark. In accordance with Danish legislation [21], [22] there were no need for an institutional review board approval for this study, as *i*) sensitive data - as defined by the Danish Data Protection Agency - was not retrieved from participants, *ii*) participants were anonymous to researchers ensuring full confidentiality, and *iii*) no experiments on humans or human biological material were carried out.

### The final data material

The final questionnaire version was used to collect data in January 2011 and was distributed to a selected sample of respondents through the internet. A total of 1,600 individuals were invited to answer and the data collection was closed when more than 800 respondents had answered. In total, 893 individuals had selected a preferred alternative from three different options (No Policy (current), (New) policy 1, (New) policy 2) in six scenarios (see example in Fig. 2). Following the completed data collection, data were scrutinised for anomalies. Some 30 respondents exhibited serial non response [23] by choosing the status quo alternative (‘No Policy’ option) with a consequential zero tax payment in all six choice sets and motivated this response pattern with a statement that “the initiatives should not be financed through income tax”. It is standard in the environmental valuation literature to assume that such respondents have not been willing to reveal their true preferences, but rather protested against the payment vehicle and thus we excluded them from the sample [24]. Likewise 27 respondents never chose the status quo and justified it by “I only considered whether the price was reflecting what I would like to contribute to a good cause”. These respondents were excluded too, as their justification indicated they did not consider the trade-off across attributes and in particular the price. Our overall results are not sensitive to the limited number of exclusions. The final sample used in the econometric modelling reported below contained 836 respondents and a total of 5,016 choices.

As often found in this kind of survey, even with a representative sample pool, the resulting samples are slightly skewed on some socio-demographic parameters. The final sample here is representative for the Danish population in terms of gender, and in terms of representation of professional and shorter educational experience, but somewhat over-represented on the longer educational experience and under-represented for the group having only primary school education. There is also the often seen issue of under-representing younger people between 18 and 39, whereas respondents over 50 are somewhat over-represented compared to the Danish population. Our main results here do however not vary much across these demographic groups, and should remain insensitive to these small imbalances.

The choice experiment design was a d-optimal design for a multinomial logit model, and had an ex ante d-error of 0.01767. It consisted of 18 choice tasks. These were allocated into three blocks, implying that each respondent had to complete six choice tasks. Furthermore, the ordering of attributes was changed for half of the respondents to avoid order-effects. The ex-post d-error for the final model was 0.00066, which is fully adequate [25].

### Methods

The choice model relies on the Random Utility Model [18] and here we report the results of a conditional logit assuming that the error terms are independently and identically drawn from an extreme value distribution. We estimated varying econometric specifications and present results here and in Supplementary Material (File S1).

As is common in the choice modelling literature [26] we assumed that the utility of a good can be described as a function of its attributes. In a choice set where alternative versions of a good are described by variation in their attributes, respondents are assumed to choose the alternative good that gives them the highest indirect utility. Since observation of utility can only be made imperfectly, the Random Utility Model provides the basis for estimation. It can be formally described as:
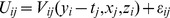
(1)


Where *U_ij_* represents individual *i*'s indirect utility from a change in bird population levels. The term *V_ij_* is deterministic and is a function of individual *i*'s income *y* reduced by a tax payment *t* for alternative *j*, the alternative's attributes *x_j_* and the individual's characteristics, *z_i_*. The error term *ε_ij_* is stochastic, i.e. it cannot be observed by the analyst. If we assume the error elements to be independently and identically drawn from an extreme value distribution, the Random Utility Model is specified as conditional logit.

If the utility function *U* is linear in its arguments and collecting all the arguments in the vector *x_ki_* for a given specific alternative *k* among the *J* choice alternatives and individual *i* choosing, we can write *U_ki_*  = *β’x_ki_*, where *β* is a vector of parameters describing alternatives in terms of a bird's population levels, conservation status in Europe, conservation status in Denmark and the price of the policy option. Using the conditional logit model, the probability of an individual *i* choosing alternative *k* over a set of alternatives *J* is given by
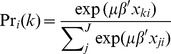
(2)where *μ* is a scale parameter which for simplicity is typically normalised to unity.

For robustness testing we used a variant of this model, which allows for describing and estimating a distribution for *β* as random parameters, hence accounting for preference heterogeneity in the population. This random parameter logit model [26], [27] describes the probabilities as integrals of the standard conditional logit function over the distribution of *β* in the *n*’th choice occasion:
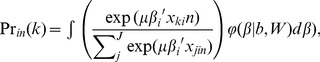
(3)


Here 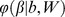
is the distribution function for *β*, with mean *b* and covariance *W*. Estimation of the likelihood function based on (3) requires that assumptions and specifications are made about which coefficients are random and the joint distribution of these coefficients. In our random parameter model we assumed all parameters except price to be normally distributed. Note that this model implies an explicit estimation of the nature of the variation in preferences across individuals, in the form of a density function. This is not to be confused with the unexplained variation in choices captured by the Gumbel error term, cf. equation (1).

The marginal value in terms of Willingness to Pay (WTP) of any attribute is computed as the negative of the coefficient on that attribute divided by the coefficient on the tax payment variable. Standard errors for the WTP estimates are estimated using the Delta Method, which is a linear approximation of the maximum likelihood function based on the variance-covariance matrix of the model parameters [28]. We estimated the models using the software Nlogit [29].

## Results

### Main effects results

**Figure 3:**
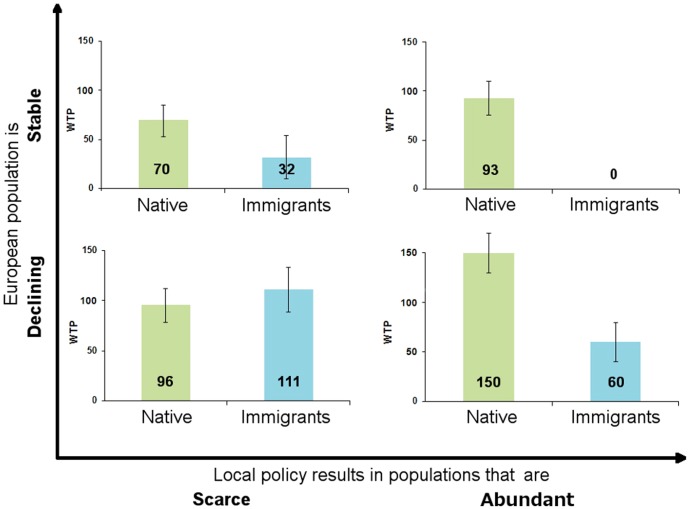
Willingness to pay for preserving birds. Peoples' mean willingness to pay (WTP) for preserving birds as a function of predicted European population (large vertical axis), local policy outcome (large horizontal axis) and whether the bird group is native or immigrating (colours). The columns show mean WTP in Euro/household and year. NB: The error bars shown indicate 95 per cent confidence intervals for WTP, and thus if the mean WTP estimates are significantly different.

People state significantly higher WTP for conserving native species in their area relative to species moving into their geographical area (Fig. 3). This is in particular true when future conservation status implies population levels to be ‘Abundant’, and when the species group is otherwise projected to have a stable population in Europe. Only when the species groups are projected to be declining at the European level are the WTP amounts for securing a future conservation status of ‘Scarce’ not statistically different between native species (96 EUR per household per year) and immigrating species (111 EUR).

Looking just at the details for native species, people state higher preferences for preserving population levels as ‘Abundant’ than ‘Scarce’ (see the details in Table 1). That people are willing to pay more for a higher quantity of the ‘good’ is a sensitivity to scope that economists would usually expect to find [30], [31]. This is particularly visible for native species declining at European level. Furthermore, people state significantly higher preferences for preserving species that are expected to have a decreasing population in Europe relative to those that are expected to be stable. While significant for both future population levels, this is especially visible for the future population level of ‘Abundant’. People thus prefer conservation actions targeted at native species which are expected to become relatively scarcer in the future at European level.

**Table 1:** Result of the conditional logit estimation.

	Policy Outcome Variables	Preference Coefficient	Std. Error	P- values	WTP (EUR)	Std. Error WTP
	Price, *β_P_*	−0.0010	0.00005	0.000	N/A	N/A
	Alternative Specific Constant	−0.2691	0.05653	0.000	−34	6.58
	**Stable** in Europe					
	Preserved as **Abundant** in DK, *β_NSA_*	0.7279	0.05069	0.000	93	8.69
**Groups of**	**Stable** in Europe					
**Native**	Preserved as **Scarce** in DK *β_NSS_*	0.5452	0.05588	0.000	70	7.92
**Species**	**Decreasing** in Europe,					
	Preserved as **Abundant** in DK, *β_NDA_*	1.1747	0.05306	0.000	150	9.96
	**Decreasing** in Europe,					
	Preserved as **Scarce** in DK, *β_NDS_*	0.7476	0.05488	0.000	96	8.54
	**Stable** in Europe					
	Preserved as **Abundant** in DK	−0.0528	0.08450	0.532	−7	10.8
**Groups of**	**Stable** in Europe					
**Immigrating**	Preserved as **Scarce** in DK	0.2473	0.08731	0.005	32	11.35
**Species**	**Decreasing** in Europe,					
	Preserved as **Abundant** in DK	0.4680	0.08343	0.000	60	10.21
	**Decreasing** in Europe,					
	Preserved as **Scarce** in DK	0.8723	0.07772	0.000	111	10.80
	*Number of observations*	*5,016*				
	*Log Likelihood Value*	−*4,577.90*				
	*Chi square*	*1,865.48*				
	*Pseudo R^2^*	*0.17*				

The preference coefficients of the *β*-vector are estimated from the logit model. The WTP estimates are obtained as the ratio of the *β* of the attribute in question relative to the *β* of price, e.g. WTP of preserving native species, with a stable European development at the level of ‘Abundant’ is: WTP  =  *β_NSA_/β_P_*. Note not all decimals shown.

Columns 3–5 show preference coefficients, standard errors and p-values for the combinations of species groups and future policy outcome in Denmark (DK) in column 1 and 2. Columns 6–7 shows the related mean Willingness to Pay (WTP) estimates (€/household and year) and standard error of this.

Looking next at WTP values for conserving immigrant species, people also have significantly higher preferences for preserving bird species expected to decrease in population levels at European level relative to those expected to remain stable at the European level. It is noteworthy, however, that people express significantly higher preferences for securing future preservation at the ‘Scarce’ level in Denmark rather than at the ‘Abundant’ level, irrespective of European population development. In fact, the parameter for a future local population at the ‘Abundant’ level for immigrant species predicted to be stable in Europe is not significantly different from zero. The parameter is even estimated as marginally negative, which may seem counterintuitive. We note that this is fully possible within the theoretical and methodological setup. The method applied here relies on relative preferences, and in particular the estimation of WTP implies relating the marginal disutility (negative) of parting with money to the marginal utility effect (which can be both positive or negative according to respondents preferences) of levels of all other attributes, i.e. of the potential environmental changes. It is perfectly standard that some environmental changes may in fact be perceived negatively by respondents, and it is usually not advisable to apply restrictions on preference parameters.

This pattern is exactly opposite to that seen for the group of native species. As an example, this implies that people are willing to pay 150 Euro per year per household for the conservation at the abundant level of species like the golden plover (*Pluvialis apricaria*), which is currently found in Denmark, but threatened at the European level. The grey-headed woodpecker (*Picus canus*) is equally threatened at the European level, but is not a breeding species in Denmark. For such species, people are only willing to pay 60 Euro for preservation at the abundant level. They are, however, willing to pay 111 Euro in total for preserving such species at the scarce level. A final comment is needed on the general level of WTP and differences across attributes. We note that the WTP levels are of a fairly similar size to other studies of enhanced biodiversity protection in Denmark [30], [32], [33].

A random parameter model allowing for distributions around the estimated parameters (cf. eq. 3) was also estimated and showed an entirely similar pattern of WTP and further revealed that preference heterogeneity was fairly low (See Table S1 in File S1).

### Supporting results for interpretation

To investigate further the robustness of the results, we undertook a more detailed examination of the pattern of responses among respondent sub-groups, notably people indicating a belief that climate change was man-made, and people who were more knowledgeable about birds – both compared to the remaining respondents. Estimations were undertaken with interaction dummies for these two respondent groups across all attributes. The analyses revealed that people indicating climate change to be man-made and people more knowledgeable about birds, had significantly higher preferences for protecting native species relative to people less knowledgeable about birds or not believing climate change to be man-made (see Table S2 and S4 in File S1). Peoples' preferences for conserving immigrant species were for most attributes not significantly different across these sub-groups, with one exception as people believing climate change to be man-made tended to prefer conserving immigrating species stable at the European level slightly less than other people.

It is possible that individuals' preferences towards the conservation of immigrant species are influenced by a concern that some of such species could be ecologically damaging. We controlled for this by informing respondents prior to the choice sets about the difference between invasive and immigrant species in order to avoid misunderstandings. This was successful, as in a follow-up question only 95 respondents (11%) stated that they thought immigrant species could be potentially negative for Danish nature, whereas 225 (27%) and 335 (43%) thought it might be positive or neutral respectively. Excluding those respondents who believe immigrant species could be potentially negative for the Danish nature does not change the pattern of the results (See Table S3 in File S1). Thus, our results do not reflect concerns about damages due to invasive species.

## Concluding Discussion

The results of this study add perspective to the discussion of the welfare economic effects of international biodiversity conservation efforts. They suggest that the values people derive from conservation outcomes go beyond the use and non-use values related to species richness and population levels of species as such, and seem to also reflect peoples' perception of the species' origins in a geographical, historical and possibly cultural sense and context.

We find that a country's citizens have a higher WTP for the conservation of birds native to their country, than for bird species moving naturally into the country – even when this runs counter to the potentially inevitable range shifts associated with climate change. The only exception is when immigrating species are potentially under pressure elsewhere in Europe, in which case people are equally willing to pay for securing their survival (at population level ‘Scarce’) for both native and immigrating species.

It is possible that many different aspects cause this pattern, and to investigate a few we estimated models where we tested whether people believing that climate change was man-made had preferences which are different from other people. Similarly, we tested whether people fairly knowledgeable about birds held different preferences for conservation actions in a climate change context than those less knowledgeable. We found in both cases that people in both of these groups (more likely to think of climate change as being due to human actions; more knowledgeable about birds) had significantly higher WTP than other people for protecting native species, whereas they had largely the same WTP as other people for protecting immigrating species. One possible interpretation of this pattern is that it reveals a willingness to help mitigate effects of climate change, reflecting the more general theory of endowment effects or loss aversion [34], [35]. This interpretation is backed up by the pattern seen for both of these groups, that are either more likely to foresee and expect the changes postulated, are more concerned about them in general, or because of their higher knowledge and acquaintance with native birds, therefore see a higher value in protecting them from the impacts in Denmark of climate change.

### Caveats and further work

As always, when eliciting peoples' views in large scale empirical studies, there is a need to consider how people interpreted core concepts of the study. In our case, we were, among other things, attentive to the potential risk of respondents confusing immigrating with invasive species, which would clearly thwart our results. We therefore designed the questionnaire with a sufficient level of information and with the possibility of identifying such a potential confusion to successfully eliminate this risk.

A crucial aspect in our study is the distinction between ‘native’ and ‘immigrating’ (non-native) species. From a biological point of view, such notions may be seen as unnecessarily static concepts, which do not take into account the natural response of species to environmental change. Many species perceived as native to any specific region may in fact only have been there for a limited time period. However, the discussion about ‘native’ species vs ‘immigrating’ or ‘non-native’ species is a very real policy discussion and underpins actual policies. For example, Danish environmental forest policy schemes encourage the use of ‘native tree species’ and discourage the use of non-native species like Norway spruce (*Picea abies*) – which might have immigrated, and certainly have certainly been “native” to Denmark in earlier time periods. The recent reintroduction of European beaver (*Castor fiber*) and the European bison (*Bison bonasus*) (both extirpated in Denmark for thousands of years) and the current re-establishment of the wolf (*Canis Lupus*), which has been absent for only 200 years, have also all spurred debates about ‘nativeness’. It seems that people's understanding of what is ‘native’ to a country may have a fairly recent and certainly quite static perception. Clearly, a deeper understanding of the roots of these debates and concepts will be illuminating and could call for other kinds of disciplines, for example by linking environmental valuation methods to environmental history [36].

Turning to conservation science, it has been pointed out that efforts targeting national objectives may reduce overall cost efficiency [37] in particular concerning the potential gains from coordination [38]–[40]. While this may be true, our results suggest that future conservation management research may be needed in coordination models, potentially based on game or bargaining theory that can integrate the potential variation among people with respect to preferences over preserving native rather than non-native species locally. Another field ahead is to look not only at the species distribution as spatially explicit, but also the demand as here expressed by WTP of people.

### Implications for conservation policy

Our findings may also have important implications for nature conservation and policy practice. The merits of geopolitical coordination in terms of the cost-effectiveness of conservation efforts [38], [39] are not contested by our study. These are the reasons why international biodiversity policies focus on coordination of efforts. However, our results show that such coordination may run counter to the preferences and welfare of citizens whose tax dollars or voluntary donations to conservation NGOs pay for many conservation actions. This matters when climate change sets species populations into motion across national boundaries.

There may be several possible responses to this discrepancy. Because our results show that people may derive a higher value from preserving native species in situ than species moving into new geographical areas, one option is that policy coordination efforts should take this into account and aim to strike a better balance between actions which minimise the cost of achieving biodiversity conservation targets with the values and concerns of the general public, and hence also allow some room for national targets for native species and habitats.

However, in situations where climate change causes significant changes in species distributions, preserving native species may be excessively costly. Thus, a second policy approach could be to communicate these risks and costs around protecting species currently viewed as “native” to voters. This implies the need to convey an understanding of the biological dynamics of species range shifts and of what can be seen as native species. In the absence of such information our results indicate a very real risk of wasting conservation efforts on potentially lost causes: Politicians may find high popular support for preserving native species under pressure, and opt for this in spite of potentially very uncertain gains. It is possible that, e.g. private and largely donation driven conservation NGOs can fill in a niche in some case, but we note that our auxiliary analyses revealed that people knowledgeable about birds had an even stronger preference for preserving native species relative to immigrants. One may speculate that this group are more likely to be active conservationists, and to give larger donations, and hence our findings also have relevance for NGO's objective setting and policies. Conservation organisations may pro-actively use social marketing concepts to promote conservation support from different typologies of donators [41].

To counteract these problems, we stress the need for sound scientific knowledge and assessments of climate change impacts on species distribution and geographical shifts of breeding range [42]. This discussion even applies to public reluctance towards optimal timing of relocating species that cannot keep pace with climate change [43]. Improved information should be able to guide the policy process and allow people to factor in the likelihood of preservation efforts to succeed in light of climate change pressure, thereby re-aligning public support with what is possible in conservation terms.

## Supporting Information

File S1 **Supplementary information.**
(DOCX)
