# Supplementary material for: Public Support for Conserving Bird Species Runs Counter to Climate Change Impacts on Their Distributions
Source: PLoS One. 2014 Jul 1;9(7):e101281. doi: 10.1371/journal.pone.0101281 (PMC4077775; doi:10.1371/journal.pone.0101281)
Supplement: File S1 — Supplementary information. (DOCX) [file pone.0101281.s001.docx]

Supplementary Materials for manuscript: **Public support for conserving bird species runs counter to climate change impacts on their distributions**

**Robustness testing of results using alternative model specification**

The results of a random parameter model are shown in Supplementary Table S1. The attributes related to bird populations are assumed normally distributed whereas price and the alternative specific constant are held fixed. The results show that preference heterogeneity is fairly low. In fact the population standard deviation is not significantly different from zero for any attribute except preserving immigrant species, which are decreasing in Europe at the level of ‘Abundant’.

**Supplementary Table S1: Random parameter model**

|  | **Policy Outcome**  **Variable** | **Preference Coefficient (random parameter)** | | **Std. Error** | **P- values** | **WTP (EUR)** | **Std. Error**  **WTP** |
| --- | --- | --- | --- | --- | --- | --- | --- |
|  | Price | -0.0011 | 0.0006 | | 0.000 | N/A |  |
|  | Alternative Specific Constant | -0.2551 | 0.0617 | | 0.000 | -30 | 6.66 |
| **Groups of**  **Native Species** | **Stable** in Europe  Preserved as **Abundant** in DK | 0.8919  (0.0953) | 0.0637  (0.2500) | | 0.000  (0.703) | 106 | 10.05 |
|  | **Stable** in Europe  Preserved as **Scarce** in DK | 0.7831  (0.0140) | 0.0757  (0.1874) | | 0.000  (0.941) | 93 | 9.98 |
|  | **Decreasing** in Europe,  Preserved as **Abundant** in DK | 1.5224  (0.0106) | 0.0801  (0.4000) | | 0.000  (0.979) | 181 | 12.24 |
|  | **Decreasing** in Europe,  Preserved as **Scarce** in DK | 1.0826  (0.0165) | 0.0783  (0.2101) | | 0.000  (0.938) | 129 | 10.57 |
| **Groups of**  **Immigrating Species** | **Stable** in Europe  Preserved as **Abundant** in DK | -0.0467  (0.0473) | 0.0879  (0.2681) | | 0.600  (0.859) | -6 | 10.47 |
|  | **Stable** in Europe  Preserved as **Scarce** in DK | 0.2933  (0.0473) | 0.0894  (0.2681) | | 0.001  (0.859) | 35 | 10.78 |
|  | **Decreasing** in Europe,  Preserved as **Abundant** in DK | 0.0344  (2.2948) | 0.1584  (0.3361) | | 0.000  (0.000) | 4 | 18.80 |
|  | **Decreasing** in Europe,  Preserved as **Scarce** in DK | 1.0265  (0.0758) | 0.0853  (0.6068) | | 0.000  (0.901) | 122 | 10.80 |
|  | *Number of observations*  *Log Likelihood Value*  *Chi square*  *Pseudo R^2^* | *5,016*  *-4,559.14*  *1,903.00*  *0.17* |  | |  |  |  |

*Note: Figures in parentheses are related to the estimated standard deviation around the random parameter, cf. equation (3).*

We also tested whether preceding bird knowledge had any influence on WTP. Before the choice experiment we asked respondents to identify five native Danish birds in a multiple choice setting. Almost 24 per cent recognised three or more of the birds and a dummy for those respondents was also interacted with the main attributes. These results can be found in Supplementary Table S2. It shows that preceding bird knowledge has a significant positive influence on WTP for the preservation of native Danish birds but no influence on the WTP for the preservation of immigrating species.

**Supplementary Table S2.** Results of the conditional logit model estimation with interaction effects of respondents knowing 3 or more of presented birds

|  | **Variable** | **Preference Coefficient** | **Std. Error** | **P-Value** | **WTP (EUR)** | **Std. Error**  **WTP** |
| --- | --- | --- | --- | --- | --- | --- |
|  | Price | -0.0010 | 0.0001 | 0.000 |  |  |
|  | Alternative Specific Constant | -0.2694 | 0.0566 | 0.000 | -34 | 7 |
| **Groups of**  **Native Species** | **Stable** in Europe  Preserved as **Abundant** in DK | 0.6069 | 0.0629 | 0.000 | 77 | 9 |
|  | *- knowledge interaction effect* | 0.3193 | 0.0985 | 0.001 | 41 | 13 |
|  | **Stable** in Europe  Preserved as **Scarce** in DK | 0.3827 | 0.0694 | 0.000 | 49 | 9 |
|  | *- knowledge interaction effect* | 0.4278 | 0.1082 | 0.000 | 54 | 14 |
|  | **Decreasing** in Europe,  Preserved as **Abundant** in DK | 1.0596 | 0.0671 | 0.000 | 135 | 11 |
|  | *- knowledge interaction effect* | 0.3123 | 0.1098 | 0.004 | 40 | 14 |
|  | **Decreasing** in Europe,  Preserved as **Scarce** in DK | 0.6068 | 0.0697 | 0.000 | 77 | 10 |
|  | *- knowledge interaction effect* | 0.3734 | 0.1133 | 0.000 | 47 | 15 |
| **Groups of**  **Immigrating Species** | **Stable** in Europe  Preserved as **Abundant** in DK | 0.0249 | 0.1040 | 0.811 | 3 | 13 |
|  | *- knowledge interaction effect* | -0.2032 | 0.1552 | 0.191 | -26 | 20 |
|  | **Stable** in Europe  Preserved as **Scarce** in DK | 0.2137 | 0.1090 | 0.050 | 27 | 14 |
|  | *- knowledge interaction effect* | 0.0808 | 0.1582 | 0.609 | 10 | 20 |
|  | **Decreasing** in Europe,  Preserved as **Abundant** in DK | 0.4590 | 0.1053 | 0.000 | 58 | 13 |
|  | *- knowledge interaction effect* | 0.0512 | 0.1644 | 0.755 | 7 | 21 |
|  | **Decreasing** in Europe,  Preserved as **Scarce** in DK | 0.9010 | 0.0918 | 0.000 | 115 | 13 |
|  | *- knowledge interaction effect* | -0.0644 | 0.1256 | 0.608 | -8 | 16 |
|  | *Number of observations*  *Log Likelihood Value*  *Chi square*  *Pseudo R^2^* | *5,016*  *-4,560.26*  *1,900.76*  *0.172* |  |  |  |  |

If respondents were wrongly perceiving immigrating species as invasive, our conclusions would be affected. Therefore we informed respondents prior to the choice experiment that immigrating birds were not invasive. In a debriefing question we asked whether they believed immigrating species to be bad for Danish nature and 11 per cent believed so. We re-estimated our model excluding these respondents in order to see if they were driving the pattern of results. Table S3 shows the result of this estimation and it shows that we find the same pattern of results when excluding this group of respondents. The conclusion is that our results are not driven by a fear of invasive species.

**Supplementary Table S3**. Results of the conditional logit model estimation without respondents who state that immigrating species are bad for nature

|  | **Variable** | **Preference**  **Coefficient** | **Std. Error** | **P-value** | **WTP**  **( EUR)** | **Std. Error WTP** |
| --- | --- | --- | --- | --- | --- | --- |
|  | Price | -0.0010 | 0.0001 | 0.000 |  |  |
|  | Alternative Specific Constant | -0.1758 | 0.0600 | 0.000 | -23 | 7 |
| **Groups of**  **Native Species** | **Stable** in Europe  Preserved as **Abundant** in DK | 0.7341 | 0.0534 | 0.000 | 96 | 10 |
|  | **Stable** in Europe  Preserved as **Scarce** in DK | 0.5115 | 0.0590 | 0.000 | 67 | 9 |
|  | **Decreasing** in Europe,  Preserved as **Abundant** in DK | 1.1330 | 0.0558 | 0.000 | 149 | 11 |
|  | **Decreasing** in Europe,  Preserved as **Scarce** in DK | 0.7065 | 0.0582 | 0.000 | 93 | 9 |
| **Groups of**  **Immigrating Species** | **Stable** in Europe  Preserved as **Abundant** in DK | -0.0540 | 0.0891 | 0.606 | -7 | 12 |
|  | **Stable** in Europe  Preserved as **Scarce** in DK | 0.1869 | 0.0928 | 0.000 | 25 | 12 |
|  | **Decreasing** in Europe,  Preserved as **Abundant** in DK | 0.4320 | 0.0878 | 0.000 | 57 | 11 |
|  | **Decreasing** in Europe,  Preserved as **Scarce** in DK | 0.7895 | 0.0821 | 0.000 | 104 | 12 |
|  | *Number of observations*  *Log Likelihood Value*  *Chi square*  *Pseudo R^2^* | *4,446*  *4,078.60*  *1,611.65*  *0.165* |  | | | |

**Supplementary Table S4.**Results of the conditional logit model estimation with interaction effects of people believing climate change is man-made. Note that the coefficients for the interaction terms are to be understood as an additional contribution for this group – to be added to the main parameter.

|  | **Variable** | **Preference Coefficient** | **Std. Error** | **P-value** | **WTP (EUR)** | **Std. Error**  **WTP** |
| --- | --- | --- | --- | --- | --- | --- |
|  | Price | -0.0010 | 0.0001 | 0.000 |  |  |
|  | Alternative Specific Constant | -0.2715 | 0.0566 | 0.000 | -35 | 7 |
| **Groups of**  **Native Species** | **Stable** in Europe  Preserved as **Abundant** in DK | 0.5890 | 0.0854 | 0.000 | 75 | 12 |
|  | *- climate change interaction effect* | 0.2129 | 0.1019 | 0.034 | 27 | 13 |
|  | **Stable** in Europe  Preserved as **Scarce** in DK | 0.3248 | 0.0960 | 0.001 | 41 | 12 |
|  | *- climate change interaction effect* | 0.3280 | 0.1133 | 0.004 | 42 | 15 |
|  | **Decreasing** in Europe,  Preserved as **Abundant** in DK | 0.9750 | 0.0926 | 0.000 | 124 | 13 |
|  | *- climate change interaction effect* | 0.2973 | 0.1129 | 0.009 | 38 | 14 |
|  | **Decreasing** in Europe,  Preserved as **Scarce** in DK | 0.5739 | 0.0973 | 0.000 | 73 | 13 |
|  | *- climate change interaction effect* | 0.2561 | 0.1179 | 0.030 | 33 | 15 |
| **Groups of**  **Immigrating Species** | **Stable** in Europe  Preserved as **Abundant** in DK | 0.1590 | 0.1415 | 0.261 | 20 | 18 |
|  | *- climate change interaction effect* | -0.3045 | 0.1639 | 0.063 | -39 | 21 |
|  | **Stable** in Europe  Preserved as **Scarce** in DK | 0.5666 | 0.1503 | 0.000 | 72 | 19 |
|  | *- climate change interaction effect* | -0.4495 | 0.1713 | 0.009 | -57 | 22 |
|  | **Decreasing** in Europe,  Preserved as **Abundant** in DK | 0.4126 | 0.1470 | 0.005 | 53 | 18 |
|  | *- climate change interaction effect* | 0.0850 | 0.1742 | 0.626 | 11 | 22 |
|  | **Decreasing** in Europe,  Preserved as **Scarce** in DK | 1.0394 | 0.1194 | 0.000 | 132 | 16 |
|  | *- climate change interaction effect* | -0.2441 | 0.1318 | 0.064 | -31 | 17 |
|  | *Number of observations*  *Log Likelihood Value*  *Chi square*  *Pseudo R^2^* | *5,106*  *-4,555.80*  *1,909.68*  *0.173* |  |  |  |  |

Overall, the findings from these models confirm the findings from the research article in PLOS ONE **Public support for conserving bird species runs counter to climate change impacts on their distributions**, namely that the value of conservation results goes beyond numbers and composition of species, but also to a very high degree reflects species origins in a geographical, historical and possibly cultural sense.

**Questionnaire**

Here follows a translated version of the Danish questionnaire made available online by using the software Survey-Xact:

| **Welcome to this survey regarding birds in nature**  **Forest & Landscape and Centre for Macroecology, evolution and climate at University of Copenhagen, are at the moment conducting a survey regarding people’s attitude towards the conditions for bird life in Denmark. The climate changes effect habitats for many animals and plants and thereby also the conditions of life for some of Europe’s birds. In the future many interests have to be taken care of when managing nature, and the viewpoints of people are important. Therefore we wish, in this survey, to get knowledge of your attitude and wishes for bird life in Denmark in the Future. Please notice: all responses are equally good.**  **The questionnaire contains a little more text than you are used to from other surveys. It is however important that you read all the information.**  **You can move forward in the survey by pressing the arrow (->) to the right on the top. There is no possibility of going back. It is an advantage to maximise the window to get a better overview. You do that by pressing the middle square in the top right corner of the window (XX) if the questionnaire is answered on a Windows PC**  **It takes approximately 20 minutes to fill out the questionnaire** |
| --- |

1) What are you doing when visiting nature?

(1) ❑ Watching animals and birds

(2) ❑ Taking photos of animals and birds

(3) ❑ Feeding wild animals and/or birds

(4) ❑ Camping

(5) ❑ Working

(6) ❑ Exercising

(7) ❑ Picking mushrooms and/or berries

(8) ❑ Picnicking

(9) ❑ Experiencing nature

(10) ❑ Biking

(11) ❑ Hunting or fishing

(12) ❑ Walking

(13) ❑ Other

**2) How many times have you been in the nature for recreation during the last 7 days? (including short and long trips outside the city)?**

**_____**

3) There are many reasons to enhance living conditions for animals in nature. Please choose the statement you agree most with.

(1) ❑ It is important, that wildlife have good living conditions, so we can enjoy them in nature.

(2) ❑ Wildlife must have good living conditions so we can rely on them being well

(3) ❑ Living conditions for wildlife should be good so our descendants can enjoy them in nature

(4) ❑ None of above (State other reason) __________

| **Recognizing birds** Now follow some questions about your knowledge of different bird species. The species are not equally easy to recognise and some of them are seen rarely by people in general. Therefore we kindly ask you to tick off only the species you know. |
| --- |

| 4.1) Do you recognise this bird?  (1) ❑ Blackbird  (2) ❑ Sparrow  (3) ❑ Starling  (4) ❑ Black woodpecker  (5) ❑ Wren  (6) ❑ No, I do not recognise it | 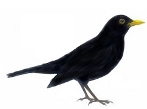 |
| --- | --- |
| 4.2) Do you recognise this bird?  (1) ❑ Grey lag-goose  (2) ❑ Mallard  (3) ❑ Shoveler  (4) ❑ Canada goose  (5) ❑ Coot  (6) ❑ No, I do not recognise it | 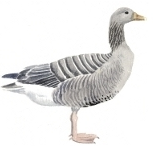 |
| 4.3) Do you recognise this bird?  (1) ❑ Grey-headed woodpecker  (2) ❑ Tree creeper  (3) ❑ Greenfinch  (4) ❑ Roller  (5) ❑ Oyster catcher  (6) ❑ No, I do not recognise it | 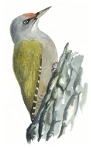 |

| 4.4) Do you recognise this bird?  (1) ❑ Shelduck  (2) ❑ Mallard  (3) ❑ Gadwall  (4) ❑ Goldeneye  (5) ❑ Great crested grebe  (6) ❑ No, I do not recognise it | 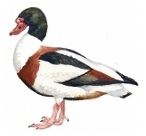 |
| --- | --- |
| 4.5) Do you recognise this bird?  (1) ❑ Golden plover  (2) ❑ Avocet  (3) ❑ Lapwing  (4) ❑ Heron  (5) ❑ Oyster catcher  (6) ❑ No, I do not recognise it | 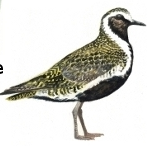 |

| **A changing nature** The Danish nature is at all times exposed to changes and is also influenced by changes in climate.  Climate changes affect the living conditions for many mammals, birds, plants and other species. In the years to come some species will experience more difficult living conditions in Denmark. They will perhaps disappear from here if we do not enhance their conditions. On the other hand, the climate changes may result in new species immigrating to Denmark. Especially if we support the living conditions for these species.  Please note, immigrating species IS NOT the same as invasive species, such as hogweed and Iberian slugs, which come here, dominate and crowd out other species. |
| --- |

5) Nature conservation policies enhancing living conditions of wildlife should prioritize...

|  | Highly agree | Somewhat agree | Neither / nor | Somewhat disagree | Highly disagree |
| --- | --- | --- | --- | --- | --- |
| ...species I know over species I don’t know | (1) ❑ | (2) ❑ | (3) ❑ | (4) ❑ | (5) ❑ |
| ...species I often see and encounter in nature over species I never see | (1) ❑ | (2) ❑ | (3) ❑ | (4) ❑ | (5) ❑ |
| ...species that are endemic to Denmark over non-endemic species | (1) ❑ | (2) ❑ | (3) ❑ | (4) ❑ | (5) ❑ |
| ... endangered species over not endangered species | (1) ❑ | (2) ❑ | (3) ❑ | (4) ❑ | (5) ❑ |

| **Initiatives for nature conservation** We can affect living conditions for animals and plants with initiatives for nature conservation. These initiatives can neutralize or support the effect of climate change but not slow down or prevent climate change itself.  Specific initiatives could be the establishment and protection of wetlands and meadows. That will support the species living in these areas, but not species living in for instance forest. Supporting these will need other initiatives. Thus there is a connection between the areas we support and the species that will benefit.  At most a policy will consist of more initiatives and the policy will thus have various effect on different species |
| --- |

6) When climate change influences the distribution of Danish species, how much do you agree with the following statements?

|  | Highly agree | Somewhat agree | Neither / nor | Somewhat disagree | Highly disagree |
| --- | --- | --- | --- | --- | --- |
| The Danish society should not use financial resources to preserve a species rare in Denmark but common in other parts of the World | (1) ❑ | (2) ❑ | (3) ❑ | (4) ❑ | (5) ❑ |
| Species that already exist in Denmark should be prioritized over species that potentially can immigrate. | (1) ❑ | (2) ❑ | (3) ❑ | (4) ❑ | (5) ❑ |

| **Choosing between different initiatives** On the following pages we ask you to choose between initiatives for nature conservation in future. It concerns different initiatives for specific bird species in Denmark and Europe which will be affected differently by climate change. We show two bird species together which will be affected in the same way by the proposed initiative. We ask you to weigh up 3 initiatives in every choice set: No change (status quo), Policy 1 and Policy 2. Every time, please choose the initiative you like best. Please note the additional yearly household income tax payment which your household will have to pay as new initiatives can be costly to the Danish society.  Results from similar surveys show that some people tend to overstate how much they are actually willing to pay for nature conservation initiatives. Please bear in mind that an additional tax payment will result in less money for you to spend on other things in daily life.  For each bird species we have described the actual status in Denmark and the **expected** development within 15 years if nothing is done (i.e. no change). Furthermore, we have described the species **expected development in Europe**. This is changed throughout the choices, depending on which birds are shown. For example, it is not always that a bird that is few in number in Denmark is few in number in Europe.  The size of the population in Denmark is divided in three categories illustrated with symbols shown below. The European development is described as either **stable** or **decreasing.**  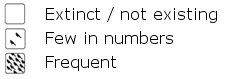 |
| --- |

**Question 7-12)**

***(Here only follows one example of a choiceset)***

**Which alternative do you prefer?**


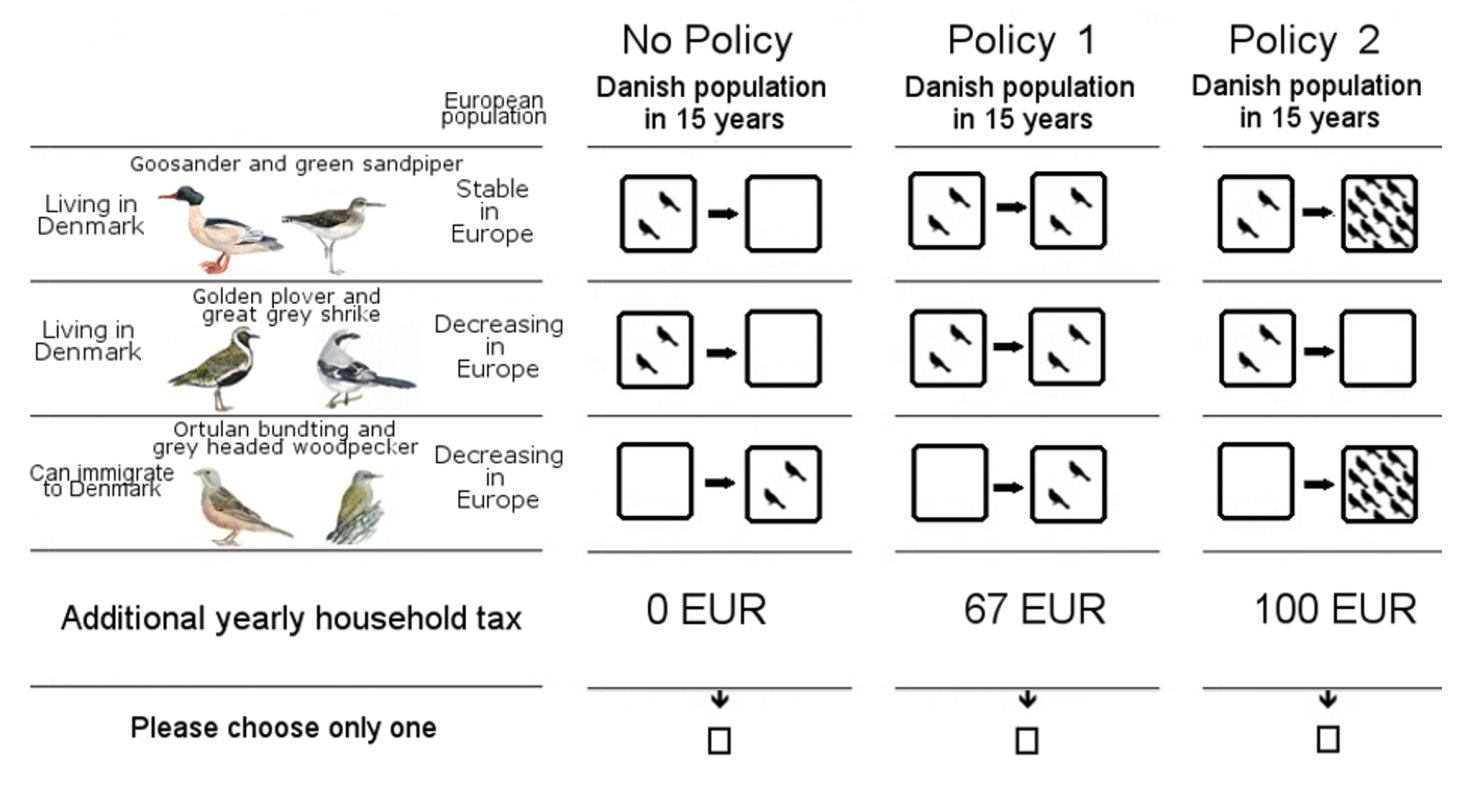


Followed by 5 more choice csets with changing levels of attributes

13) It is not obvious how initiatives will affect bird species’ living conditions.
When you choose between alternatives, did you assume the alternatives’ ability to secure...

|  | Very uncertain | Rather uncertain | Rather certain | Very certain | Don’t know |
| --- | --- | --- | --- | --- | --- |
| ... Danish species frequent in numbers from extinction was ... | (1) ❑ | (2) ❑ | (3) ❑ | (4) ❑ | (5) ❑ |
| ...new species can immigrate and become frequent was... | (1) ❑ | (2) ❑ | (3) ❑ | (4) ❑ | (5) ❑ |
| ... that endangered Danish species can survive was... | (1) ❑ | (2) ❑ | (3) ❑ | (4) ❑ | (5) ❑ |
| ...that endangered Danish species can become frequent was... | (1) ❑ | (2) ❑ | (3) ❑ | (4) ❑ | (5) ❑ |

| **Outcome uncertainty In the following six choice sets we have added a line about the probability that the initiative being provided, will work.**  **Please take the information into account when choosing the alternative you prefer.** |
| --- |

**14-19)**

Example of choice set with outcome uncertainty

Which alternative do you prefer?


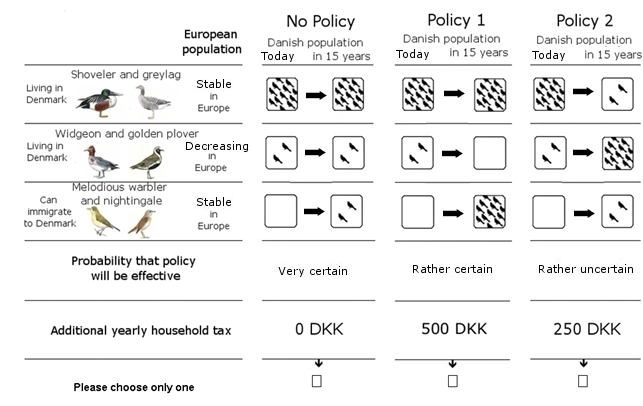


Followed by 5 more choice cards with changing levels of attributes

20) In every one of the previous 2 x 6 choice sets you chose the alternative ‘no change’. Please state the most important reason below. [if q7-12 and q13-q19 all are status quo]

(1) ❑ Birdlife in Denmark doesn’t mean anything to me

(2) ❑. Nature must run its course without our intervention

(3) ❑ The initiatives should not be financed through income tax

(4) ❑ The trade-off between the different attributes made the no change situation the best alternative in all choice sets

(5) ❑ I could not afford any of the proposed initiatives

(6) ❑ The choices were difficult to relate to

(7) ❑ Other, please state __________

21) In every one of the previous 2x6 choice sets you never chose the ‘no change’ alternative. Please state the most important reason below. [if none of q7-12 and q13-q19 are status quo]

(1) ❑ I only considered whether the price was reflecting what I would contribute to a good cause for nature

(3) ❑ The trade-off between the different attributes made always the no change situation the worst alternative.

(4) ❑ Other, please state __________

22) To which degree did you presume the following in the 2x6 choice sets?
I made my choices presuming that the changes each alternative caused...

|  | To a high degree | To some degree | To a lesser degree | Not at all | Don’t know |
| --- | --- | --- | --- | --- | --- |
| ... was worth the payment for me and my household | (1) ❑ | (2) ❑ | (3) ❑ | (4) ❑ | (5) ❑ |
| ...was a fair ambition for society given the many other problems that have to be managed | (1) ❑ | (2) ❑ | (3) ❑ | (4) ❑ | (5) ❑ |

23) Were there any attributes you did not consider in the 2x6 choice sets?

(1) ❑ The yearly additional household tax payment

(2) ❑ The different species of birds

(3) ❑ The population size and development of birds in **Denmark**

(4) ❑ The population development of birds in **Europe**

(6) ❑ The probability of provision (only the last 6 choice sets)

(5) ❑ I took everything into account

24) How important did you find the following attributes in the 2x6 choice sets? (Please rank the attributes from 1 to 5, 1 being the most important and 5 the least important)

|  |  |
| --- | --- |
| Bird species | __________ |
| Birds’ **future** population size in **Denmark.** | __________ |
| The annual tax payment for your house hold | __________ |
| Bird populations development in / The population size and development of birds in **Denmark** | __________ |
| Birds present population size in **Denmark** | __________ |

25) The information about species development under climate change relies on best possible expert judgement
In case no new initiatives are proposed for the species, do you then consider it likely that

|  | Not likely | Less likely | Likely | Very likely | Don’t know |
| --- | --- | --- | --- | --- | --- |
| ...frequent species will disappear from Denmark? | (1) ❑ | (2) ❑ | (3) ❑ | (4) ❑ | (6) ❑ |
| ...frequent species will be few in numbers in Denmark? | (1) ❑ | (2) ❑ | (3) ❑ | (4) ❑ | (6) ❑ |
| ... new species will immigrate to Denmark and become frequent? | (1) ❑ | (2) ❑ | (3) ❑ | (4) ❑ | (6) ❑ |

26) How do you appraise the Danish society’s present effort for nature and thereby the living conditions for animal and plants?

(1) ❑ The society’s effort in nature conservation is too high

(2) ❑ The society’s effort in nature conservation is adequate

(3) ❑ The society’s effort in nature conservation is too small

(4) ❑ Don’t know

27) How do you look upon new species immigrating to Denmark if the species in Europe is...

|  | Good for nature in Denmark | Neither / nor | Bad for nature in Denmark | Don’t know |
| --- | --- | --- | --- | --- |
| ...stable? | (1) ❑ | (2) ❑ | (3) ❑ | (4) ❑ |
| ... decreasing? | (1) ❑ | (2) ❑ | (3) ❑ | (4) ❑ |

**28) What do you think about society using financial resources for the benefit of nature?**

|  | Yes | No | Don’t know |
| --- | --- | --- | --- |
| Society should be able to afford to secure living conditions for species that already exists in Denmark | (1) ❑ | (2) ❑ | (3) ❑ |
| Society should be able to afford to secure living conditions for species that potentially will immigrate to Denmark | (1) ❑ | (2) ❑ | (3) ❑ |
| I am worried whether society can afford to prioritize living conditions for animal species, and I think less ambitious goals would be sufficient | (1) ❑ | (2) ❑ | (3) ❑ |
| Society should only use resources to secure just a minimal viable population | (1) ❑ | (2) ❑ | (3) ❑ |

29) Climate change is a huge part of public debate today. How much do you agree with the following statements?

|  | Highly agree | Somewhat agree | Neither / nor | Somewhat disagree | Highly disagree |
| --- | --- | --- | --- | --- | --- |
| Climate change is most likely human made | (1) ❑ | (2) ❑ | (3) ❑ | (4) ❑ | (5) ❑ |
| Climate change will affect our ways of life in the following decades | (1) ❑ | (2) ❑ | (3) ❑ | (4) ❑ | (5) ❑ |
| Climate has changes in the last decades | (1) ❑ | (2) ❑ | (3) ❑ | (4) ❑ | (5) ❑ |

| Finally we ask you to answer a few questions about yourself. We do this in order to compare answers to the previous questions from different parts of the population. We would like to stress that all answers are treated confidently, and reported results can never be traced back to any individuals. |
| --- |

30)

| How many persons at 18 years of age and above are in your house hold (including you)? | __________ |
| --- | --- |
| How many children below 18 years of age are in your household? | __________ |

31) Have you changed behaviour due to climate change (less use of car, or plane as mode of travel)

(1) ❑ Yes

(2) ❑ No

32) Are you a member of one or more of the following recreational or environmental organisations?

(1) ❑ Danish Nature Conservation Association

(2) ❑ WWF

(3) ❑ Danish Ornithologist Association

(4) ❑ Hunting organisation

(5) ❑ Fishing association

(6) ❑ Animal Protection Association

(7) ❑ Other associations or organisations with interest in nature __________

33) How is your present housing conditions?

(1) ❑ Self owned house

(2) ❑ Self owned apartment

(3) ❑ House of apartment under a multi-ownership scheme

(4) ❑ Rental house/apartment

(5) ❑ Other __________

34) What is your house holds total yearly income before tax?

(1) ❑ Under 100,000 DKK

(2) ❑ 100,000 – 199,999 DKK

(3) ❑ 200,000 – 299,999 DKK

(4) ❑ 300,000 – 399,999 DKK

(5) ❑ 400,000 – 499,999 DKK

(6) ❑ 500,000 – 599,999 DKK

(7) ❑ 600,000 – 699,999 DKK

(8) ❑ 700,000 – 799,999 DKK

(9) ❑ 800,000 – 899,999 DKK

(10) ❑ 900,000 DKK or more

35) What is your expectation about your household’s total income before tax in 10 years?

(1) ❑ Less than today

(2) ❑ Same as today

(3) ❑ Higher than today

36) How much higher do you expect your house holds total income before tax to be in 10 years?

[If q35=3]

(1) ❑ 0-20,000 DKK more per year before tax

(2) ❑ 20,000 – 50,000 DKK more per year before tax

(3) ❑ 50,000 – 100,000 DKK more per year before tax

(4) ❑ 100,000 – 200,000 DKK more per year before tax

(5) ❑ 200,000 – 400,000 DKK more per year before tax

(6) ❑ More than 400,000 DKK per year before tax

37) Why do you expect a higher income for your household in 10 years?

[If q35=3]

(1) ❑ Salary increases in general

(2) ❑ Change of job to something better paid for one or more members of my household

(3) ❑ . One or more members of my households are on leave or working part time at the moment and expect to work more in 10 years’ time

(4) ❑ One or more members of my household are currently studying and expect to be working in 10 years’ time

(5) ❑ One or more members of my household are currently unemployed and expect to employed in 10 years’ time

(6) ❑ Other, please state __________

38) How much less do you expect your household’s total income to be in 10 year?

[If q35=1]

(1) ❑ 0-20,000 DKK less per year before tax

(2) ❑ 20,000 – 50,000 DKK less per year before tax

(3) ❑ 50,000 – 100,000 DKK less per year before tax

(4) ❑ 100,000 – 200,000 DKK less per year before tax

(5) ❑ 200,000 – 400,000 DKK less per year before tax

(6) ❑ Less than 400,000 DKK per year before tax

39) Why do you expect less total income for your household in 10 years?

[If q35=1]

(1) ❑ One or more members of my household are expected to be unemployed in 10 years

(2) ❑ One or more members of my household are expected to be working part time or be retired in 10 years

(3) ❑ Other, please state: __________

40) How do you experience your household’s economic situation (income, consumption possibilities, wealth) compared to

|  | I **experience** my economic situation as... | | | |
| --- | --- | --- | --- | --- |
|  | Worse | Same | Better | Don\t know |
| Friends and acquaintances | (1) ❑ | (2) ❑ | (3) ❑ | (4) ❑ |
| Family | (1) ❑ | (2) ❑ | (3) ❑ | (4) ❑ |
| Neighbours | (1) ❑ | (2) ❑ | (3) ❑ | (4) ❑ |

If you have any comments to the survey please feel free to note them here

_____________________________________________________________
_____________________________________________________________
_____________________________________________________________
_____________________________________________________________
_____________________________________________________________
_____________________________________________________________

| **THANK YOU VERY MUCH FOR YOUR ASSISTANCE!**  Results of the bird quiz  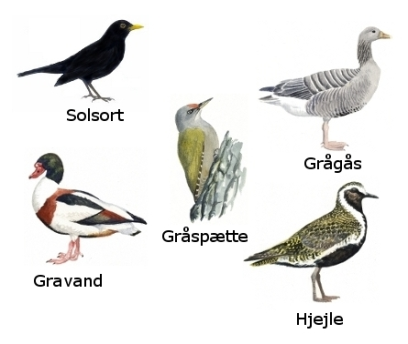 |
| --- |
